# Is Acquired Disgust More Difficult to Extinguish Than Acquired Fear? an Event-Related Potential Study

**DOI:** 10.3389/fpsyg.2021.687779

**Published:** 2021-11-19

**Authors:** Qing Zeng, Lishan Lv, Xifu Zheng

**Affiliations:** ^1^School of Marxism, Jinan University, Guangzhou, China; ^2^School of Foreign Studies, South China Agricultural University, Guangzhou, China; ^3^School of Psychology, South China Normal University, Guangzhou, China

**Keywords:** extinction, conditioned response, disgust, fear, ERP

## Abstract

This study used the classical conditioned acquisition and extinction paradigm to compare which of the two emotions, acquired disgust and acquired fear, was more difficult to extinguish, based on behavioral assessments and the event-related potential (ERP) technique. Behavioral assessments revealed that, following successful conditioned extinction, acquired disgust was more difficult to extinguish. The ERP results showed that, at the early stage of P1, the amplitude of conditioned fear was significantly smaller than that of conditioned disgust, and both were significantly different from the amplitude under neutral conditions; at the middle stage of N2, the difference between the amplitudes of conditioned disgust and conditioned fear disappeared, but they were still significantly different from the amplitudes of conditioned neutral stimuli; at the late stage of P3, the difference between conditioned disgust and conditioned neutral stimuli disappeared, but the difference between conditioned fear and neutral stimuli remained, suggesting that acquired fear was more difficult to extinguish than acquired disgust in terms of how the brain works.

## Introduction

Fear and disgust are intense and unpleasant emotions. They share some common features: both represent a central threat emotion in psychopathology, underlying distress, and avoidant behavior toward biological and psychological contamination and violation (Rosen and Schulkin, 1988). However, they are more often independent of each other. Moreover, fear is the typical emotion of an anxiety disorder (Woody and Teachman, 2000), while disgust is a characteristic trait that may distinguish certain phobias from other anxiety disorders (Rosen and Schulkin, 1988).

There have been many studies on how the neural mechanism works in the extinction of conditioned fear, and evidence showed that the extinction of conditioned disgust differs from the extinction of conditioned fear. However, few people have studied the extinction of conditioned disgust.

For example, some researches showed that during extinction, participants continued to evaluate the CS+ as more disgusting than the CS−, whereas distress and fear-related emotional ratings attenuated on electrodermal and evaluative responses (Olatunji et al., 2007; Ludvik et al., 2015). Clinical studies have found that, when individuals are exposed to a fear-related stimulus, their disgust responses are similar to their fear responses (Olatunji et al., 2009; Armstrong et al., 2019). However, self-reported disgust ratings were used as the main indicators in these studies. It is, therefore, necessary to adopt new and more objective methods to study disgust extinction.

Like conditioned fear, the research paradigm for the extinction of conditioned disgust is Pavlov’s classical model for the extinction of conditioned responses: In extinction learning, when a previously conditioned stimulus (CS+) is no longer followed by an unconditioned stimulus (US) and another conditioned stimulus (CS−) is never followed by an unconditioned stimulus, repeating this process several times will lead to the extinction of previously conditioned responses and to the formation of extinction recall (Milad and Quirk, 2012).

This study used behavioral assessments and the event-related potential (ERP) technique to examine the differences between the extinction of conditioned disgust and that of conditioned fear in humans based on the classically conditioned response paradigm. In previous ERP studies on conditioned extinction, the researchers believed that N2 mainly reflected the nerve conduction at the middle and late stages of conditioned associative learning, while the P3/LPP component mainly reflected the extent to which learning and extinction were realized at the late stage (Sun and Zheng, 2014; Li et al., 2019). Studies have shown that cognitive processing at the early stage is a bottom-up automatic process. Therefore, at the early stage of conditioned extinction, the recall of the CS-US association formed at the acquisition stage should be still working. However, as processing progressed toward top-down cognitive processing, following conditioned extinction training, the average amplitude elicited by conditioned disgust and fear stimuli was similar to that elicited by conditioned neutral stimuli. Some researchers believed that the level of conditioned fear decreases as extinction progresses, but conditioned disgust remains at a high level after extinction (Schindler et al., 2020). In this study, our hypotheses were as follows: (1) The average amplitudes elicited by conditioned disgust and fear stimuli were significantly different from those elicited by conditioned neutral stimuli in the case of early components; (2) The two types of conditioned negative stimuli were significantly different; and (3) At the late stage, brainwaves in the extinction of conditioned disgust and those in the extinction of conditioned fear were still different and that the amplitude of conditioned disgust was larger.

## Materials and Methods

### Participants

Twenty-eight college students (15 male) participated in the experiment (age 18–26 years, with an average of 20.96). All of them passed the Anxiety Inventory, Beck Depression Inventory, and Disgust Scale assessments before the experiment. They were all right-handed, had normal visual acuity (whether corrected or not), and were free from physical illnesses or mental disorders. They participated in the experiment by self-registration, and none of them had participated in similar experiments before. They were informed that the experiment might include several aversive images, and they were allowed to terminate the experiment at any time without any penalty. All of them provided their informed consent before the experiment and received RMB 50 in compensation afterward.

### Materials

#### Conditioned Stimuli

The three conditioned stimuli were photographs of a square, a circle, and a polygon (Zeng et al., 2015).

#### Unconditioned Stimuli

A total of 135 images were selected from the International Affective Picture Set (IAPS; Libkuman et al., 2007) to convey fear, disgust, or neutral content. Independent ratings of these images on valence, arousal, fear, and disgust dimensions confirmed that the picture sets evoked the intended emotions (Zeng and Zheng, 2016), including 45 eliciting disgust, 45 eliciting fear, and 45 neutral images1.^1^ The disgust pictures depicted dead animals, dirty toilets, contaminated food, maggots, and disgusting actions (e.g., vomiting). The fear pictures depicted aggressive animals, pointed guns, violent actions, and dangerous scenes (e.g., riots and car accidents). Pictures that were believed to generate feelings of both fear and disgust (e.g., mutilation and spiders) were discarded. The neutral pictures depicted household objects, peaceful scenes, and simple everyday actions (e.g., typing and reading).

### Procedure

The experiment took place in a dimly lit, sound-attenuated room. The participants were informed beforehand that a series of geometric figures and images would be presented on the screen. What they needed to do during the experiment was to look at the screen carefully and pay attention to the relationships between the geometric figures and the images. And the relationships between geometric shape and image type were counterbalanced across participants. They were presented with a practice block of nine trials to familiarize them with the task, followed by the formal experiment. The participants were required to view the images passively. To ensure that the participants did indeed learn the CS-US relationship, participants were asked to rate the valence, arousal, degree of fear, and degree of disgust of the CS images by means of a 9-point rating scale for three times: pre-acquisition, post-acquisition/pre-extinction, and post-extinction.

#### Conditioning Procedure

Every trial started with acquisition training. A red fixation cross was presented at the center of the screen for 500 ms, and participants were reminded to look at the screen. This was followed by a blank screen with a duration varying between 600 and 1,000 ms. The CS (a square, a circle, or a polygon) was then presented at the center of the screen for 1,000 ms, followed by the US (a disgust-eliciting, a fear-eliciting, or a neutral image) for 1,000 ms. The trial ended with a blank screen with a duration of 500 ms. Participants viewed two blocks of pictures, and each block had 135 trials, including 45 trials of each stimulus pair intermixed in a random order. After rating the CS images, extinction training began. The process at this stage was the same as that at the acquisition stage, except that no disgust-eliciting or fear-eliciting image was presented after a CS or after a neutral image, and a black screen was presented for 1,000 ms instead.

### Electroencephalographic Data Recording

The Electroencephalographic (EEG) was recorded using a Brain Products EEG recorder and 64 tin electrodes according to the international 10–20 system. As three electrodes were damaged, the data were actually recorded using 59 electrodes. All electrode impedances were reduced to 5 kΩ. A band-pass filter from 0.01 to 100 Hz was applied, and the EEG and EOG were consistently sampled at 500 Hz/channel. The digital filter for offline analysis was low-pass 30 Hz (24 dB/octave), which automatically corrected EOG artifacts. Trials with EOG voltage exceeding ±100 μV were excluded from the average, and the resulting ERP waveform was submitted to a phase-free 0.1–30 Hz digital filter. The EEG activity presented in geometric figures was averaged. In the experiment, three types of EEG were obtained from each participant: a CS followed by a fear-eliciting image (CS-fear, “CSf”), a CS followed by a disgust-eliciting image (CS-disgust, “CSd”), and a CS followed by a neutral image (CS-neutral, “CSn”). The time course of the EEG analysis was 1,200 ms, and the baseline was 200 ms before a CS was presented.

### Electroencephalographic Data Analysis

On average, about 84 segments for each condition were used for averaging, with 12.2% of the trials excluded from the analysis. Based on previous studies and the purpose of this study, we analyzed the average amplitudes of the P1 component (time window: 80–120 ms), the N2 component (time window: 260–340 ms), and the P3 component (time window: 350–420 ms). Following previous studies (Ugland et al., 2012), 12 electrode positions in the parieto-occipital region were chosen for the P1 component, including six (O1, PO7, PO3, P5, P3, and P1) on the left and six (O2, PO6, PO4, P6, P4, and P12) on the right, while 18 electrode positions in the frontal region and the central region were chosen for the N2 and P3 components, including nine (C5, C3, C1, FC5, FC3, FC1, F5, F3, and F1) on the left and nine (C6, C4, C2, FC6, FC4, FC2, F6, F4, and F2) on the right. Two-way 3 (types of CSs: CSd, CSf, and CSn) × 2 (hemispheres: left and right) repeated-measures ANOVAs were conducted on the average amplitudes of these electrode positions within a certain time window. The arithmetic mean of the average amplitudes of all the electrodes in each hemisphere within the same time window was taken as the dependent variable. All analyses were performed using SPSS 20.0, and the Greenhouse-Geisser method was used to correct the *p*-values and degrees of freedom in the ANOVA.

## Results

### Behavioral Results

We used one-way (types of CS: CSd, CSf, and CSn) repeated-measures ANOVAs.

#### Habituation

Prior to conditioning, no significant differences in valence ratings [*F*(2,54) = 0.37, *p* = 0.69], arousal ratings [*F*(2,54) = 0.81, *p* = 0.452], disgust ratings [*F*(2,54) = 2.02, *p* = 0.142], and fear ratings [*F*(2,54) = 0.59, *p* = 0.559] were observed between the three types of CSs, suggesting that there were no significant differences at the initial stage.

#### Conditioning

The results showed significant differences between the valence ratings of the three types of CSs [*F*(2,54) = 9.57, *p* < 0.001, η2 = 0.262]. Further multiple comparisons revealed no significant difference between the valence ratings of CSd and CSf (*p* = 0.78), and both had higher negative values than CSn (ps < 0.05). Significant differences were observed in arousal ratings between the three types [*F*(2,54) = 7.18, *p* = 0.002, η2 = 0.210]. Further multiple comparisons revealed no significant difference in arousal ratings between CSd and CSf (*p* = 0.41); however, their arousal ratings were significantly higher than that of CSn (ps < 0.05). In terms of disgust ratings, significant differences were also observed between the three types of CS [*F*(2,54) = 35.58, *p* < 0.001, η2 = 0.391]. Further multiple comparisons revealed that the degree of disgust elicited by CSd was significantly higher than that elicited by CSf (*p* = 0.003) and CSn (*p* < 0.001); at the same time, the degree of disgust elicited by CSf was significantly higher than that elicited by CSn (*p* < 0.001). In terms of fear ratings, significant differences were observed between the three types of CSs [*F*(2,54) = 52.22, *p* < 0.001, η2 = 0.659]. Further multiple comparisons revealed that the degree of fear elicited by CSf was significantly higher than that elicited by CSd (*p* < 0.001) and CSn (*p* < 0.001); in addition, the degree of disgust elicited by CSd was significantly higher than that elicited by CSn (*p* < 0.001). The results showed that the participants successfully learned the relationships between the three types of geometric figures and images. In other words, conditioned responses were successfully elicited from them.

#### Extinction

The results showed that there were significant differences in valence ratings between the three types of CS [*F*(2,54) = 2.31, *p* = 0.11, η2 = 0.008]; the differences in arousal ratings were not significant [*F*(2,54) = 0.21, *p* = 0.812, η2 = 0.008]. In terms of disgust ratings, significant differences were observed between the three types of CSs [*F*(2,54) = 9.05, *p* < 0.001, η2 = 0.546]. Further multiple comparisons revealed no significant difference in disgust ratings between CSd and CSf (*p* = 0.485), but their disgust ratings were significantly higher than that elicited by CSn (ps < 0.004). In terms of fear ratings, no significant difference was observed between the three types of CS [*F*(2,54) = 2.52, *p* = 0.090, η2 = 0.09].

### Event-Related Potential Data

During the conditioning phase, we found significantly enhanced conditioned responses to the CS+ as compared with the CS− (see Zeng and Zheng, 2018).

P1: Based on repeated-measures ANOVAs conducted on the average of P1 amplitudes elicited by different CS pictures during extinction, there was a significant main effect of CS type [*F*(2,54) = 5.24, *p* = 0.008, η2 = 0.17]. Further multiple comparisons revealed that the P1 amplitude elicited by CSd (*M* = 2.37 ± 0.28 μV) was significantly higher than that elicited by CSf (*M* = 1.45 ± 0.29 μV; *p* = 0.001) but was not significantly different from that elicited by CSn (*p* = 0.64); the P1 amplitude elicited by CSn (*M* = 2.21 ± 0.33 μV) was significantly higher than that elicited by CSf (*p* = 0.031). There was a significant main effect of laterality [*F*(1,27) = 4.31, *p* = 0.48, η2 = 0.14]. The average amplitude of the right hemisphere (*M* = 2.25 ± 0.28 μV) was significantly higher than that of the left hemisphere (*M* = 1.77 ± 0.26 μV; *p* = 0.048). No significant interaction existed between CS type and laterality [*F*(2,54) = 2.04, *p* = 0.14].

N2: Based on repeated-measures ANOVAs conducted on the average of N2 amplitudes elicited by different CS pictures during extinction, there was a significant main effect of CS type [*F*(2,54) = 5.43, *p* = 0.007, η2 = 0.17]. Further multiple comparisons revealed that the amplitude of the N2 component elicited by CSd (*M* = 0.78 ± 0.41 μV) was significantly smaller than that elicited by CSn (*M* = 1.54 ± 0.38 μV; *p* = 0.003); the amplitude of the N2 component elicited by CSf (*M* = 0.85 ± 0.33 μV) was significantly smaller than that elicited by CSn (*p* = 0.013); no significant difference was observed between the average amplitudes elicited by CSd and CSf (*p* = 0.794). The main effect of laterality was not significant [*F*(1,27) = 0.02, *p* = 0.895]. No significant interaction existed between CS type and laterality [*F*(2,54) = 0.73, *p* = 0.488].

**FIGURE 1:**
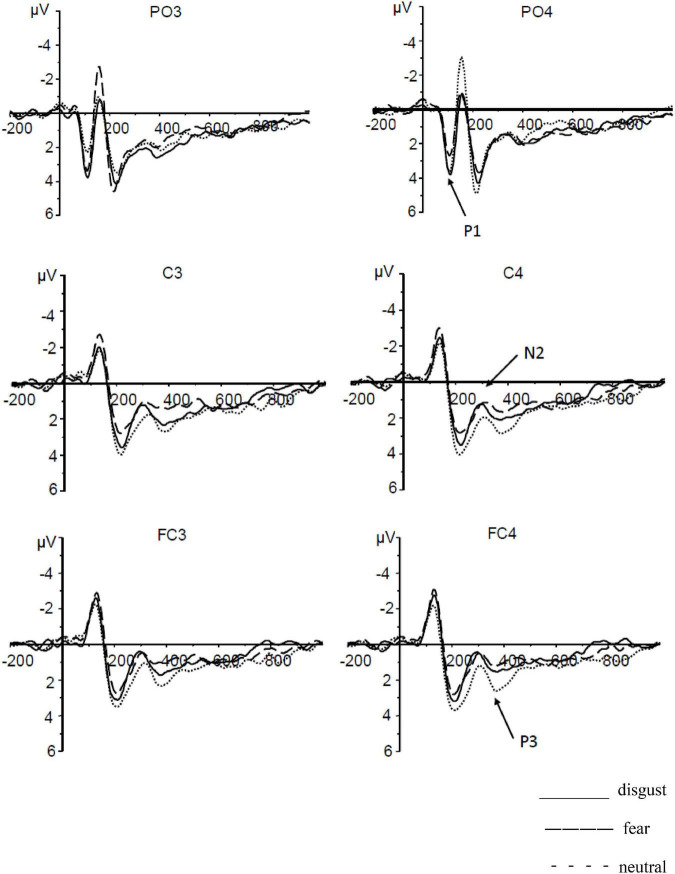
The average amplitudes of P1, N2, and P3 in conditioned disgust and fear extinction.

P3: Based on repeated-measures ANOVAs conducted on the average of P3 amplitudes elicited by different CS pictures during extinction, there was a significant main effect of CS type [*F*(2,54) = 7.16, *p* = 0.002, η2 = 0.21]. Further multiple comparisons revealed no significant difference between the P3 amplitude elicited by CSd (*M* = 1.47 ± 0.49 μV) and that elicited by CSn (*M* = 2.16 ± 0.47 μV; *p* = 0.088); however, the P3 amplitude elicited by CSf (*M* = 1.00 ± 0.47 μV) was significantly smaller than that elicited by CSn (*p* = 0.004). No significant difference was observed between the average amplitudes elicited by CSd and CSf (*p* = 0.13). The main effect of laterality was not significant [*F*(1,27) = 0.02, *p* = 0.895]. No significant interaction existed between CS type and laterality [*F*(2,54) = 2.50, *p* = 0.091] (see Figure 1).

## Discussion

This study examines the differences between the extinction of conditioned disgust and that of conditioned fear from behavioral and cognitive neurological perspectives with a focus on comparing the differences in the course of extinction. According to the ERP results, we found that disgust, following conditioned extinction training, was more difficult to extinguish than fear at the behavioral level. Moreover, at the late stage of P3, the difference between conditioned disgust and conditioned neutral stimuli disappeared, while the difference between conditioned fear and neutral stimuli remained, suggesting that fear was more difficult to extinguish than disgust.

At the behavioral level, following conditioned extinction training, there was no significant difference in valence ratings, arousal ratings, and fear ratings between the three types of images, suggesting successful extinction. However, this is inconsistent with the results of previous studies (Dwyer et al., 2007; Blechert et al., 2008; Moran et al., 2020), mainly due to the use of evaluative responses in behavioral evaluation in this study. According to the propositional model, valence ratings change in evaluative responses, because individuals consciously form a proposition that there is a pairing of CS-US and decide whether to prefer the CS on the condition of the CS-US proposition (Charash and McKay, 2009; Knowles et al., 2018). It was also reported that, with enough trials, immediate experiences of the valence of the CS will eventually be extinguished (Panitz et al., 2019). This study used the ERP technique. In order to have a sufficient number of trials for averaging, 90 extinction trials were conducted in contrast to 8–10 trials in studies in general (Ludvik et al., 2015). This is significantly higher than the number of trials used in other behavioral studies to ensure sufficient trials for participants to confirm whether the CS-noUS proposition is true. As a result, the difference in evaluation indicators between the CS+ and the CS− disappeared after extinction training was completed. Interestingly, though so many extinction trials were performed, subjective disgust ratings were still significantly different from the CS−; but regarding subjective fear ratings, the difference between the CS+ and the CS− disappeared, suggesting that evaluative disgust was more difficult to extinguish. The behavioral results of this study also support the conclusion that conditioned disgust inhibits extinction. In other words, although exposure therapy can effectively reduce the fear response, the results of self-evaluations, behavioral observations, visual avoidance tasks, and affective priming tasks (Mason and Richardson, 2010; Engelhard et al., 2014) showed that exposure therapy was not as effective as in disgust extinction (Olatunji et al., 2007).

Disgust is considered to be associated with two maladaptive cognitive processes that can potentially facilitate acquisition and impede extinction. One of the processes is referred to as the “contagion” or “once in contact, always in contact” principle, which suggests that contact with or proximity to a disgusting object can cause its properties to be permanently transferred to stimuli associated with it (Basanovic et al., 2017).

The ERP results indicated that the amplitude of conditioned fear was significantly smaller than that of conditioned disgust at the early stage of brain components, and both were significantly different from the results under neutral conditions. It was very similar to the amplitude at the late stage of acquisition (i.e., the processing stage), suggesting that all of the participants were successfully emotionally conditioned (Zeng and Zheng, 2018). It also showed that more attention was paid to the CS+ than to the CS− at the early extinction phase. At the middle stage of N2, the amplitude of conditioned disgust decreased and its difference from the amplitude of conditioned fear disappeared, but it was still significantly different from the amplitudes of conditioned neutral stimuli; the N2 component reflects the processing of information of an individual including the identification, classification, and evaluation of stimuli (Lin et al., 2017). It also showed that more attention was paid to the CS+ than to the CS− at the early extinction phase, suggesting that at the middle of the extinction phase, decreased attention is paid to conditioned disgust and the extinction of conditioned disgust begins. At the late stage of P3, the difference in amplitude between conditioned disgust and conditioned neutral stimuli disappeared, but the difference between conditioned fear and neutral stimuli remained. The P3 amplitude is considered to be an important indicator of the amount of attention paid (Milne et al., 2013; Drollette et al., 2014). A study showed that the higher the amount of attention paid to the target stimulus, the bigger the P3 amplitude is Hajcak et al. (2010), suggesting that fear is more difficult to extinguish than disgust. In general, attention control plays an important role in terms of how the neural mechanism processes information in the extinction of conditioned disgust and fear. Attention is mainly processed in a bottom-up way at the early stage of extinction. At this stage, the conditioned effects of acquisition cause more attention to be unconsciously paid to the CS+; at the late stage, attention is controlled in a top-down manner where a CS-noUS association arises and less attention is paid to the CS+ as extinction progresses.

Another interesting finding is that, despite successful extinction, the amplitude elicited by the CS− was larger than that elicited by the CS+ either at the early stage or at the late stage of the extinction, which is inconsistent with the result of a previous study (Tabbert et al., 2006; Milad and Rauch, 2007; Milad et al., 2010). There are two main reasons: First, the large amplitude elicited by the CS− might be due to conflicting expectations which led to increased attention/arousal. In other words, the participants associated the CS+ image with the negative result at the acquisition stage, but the CS+ was no longer followed by the negative result at the extinction stage where participants might expect the CS− to be a new danger signal, namely, a sudden, unexpected association (Schiller et al., 2008; Schiller and Delgado, 2010). Therefore, as a result of increased attention due to uncertainty of participants about this sudden association, the amplitude elicited by the CS− was larger than that elicited by the CS+ (Klucken et al., 2013). Although most studies have suggested that there is greater brain activity under the CS+, there are also studies that have found greater brain activity under the CS− with similar findings with regard to skin conductance and hemodynamic indicators. For example, Phelps et al. (2004) found greater brain activity in amygdala and ventromedial prefrontal cortex under the CS− than that under the CS+ in extinction learning (Phelps et al., 2004; Koizumi et al., 2016). Ugland et al. (2012) found stronger skin conductance responses under the CS− than that under the CS+ in extinction learning. Second, substantial differences in the choice of CS (e.g., using pictures as CS vs. using neutral words as CS) and differences in the experimental protocol (duration, conditioning, and extinction trials, etc.) could provide explanations for some of these contrary results. The potential explanations given above certainly need to be supported by further experimental evidence.

The results of this study suggest that exposure therapy, which is explicitly based on models of extinction, may not affect fear reactions. Therefore, the efficacy of treatment may be compromised in cases where fear is primary. As such, patients are likely to remain distressed and functionally impaired. However, the residual fear is likely to lead to relapse in the long term. Future research should identify and assess potential strategies to reduce this resistance to the extinction of learned fear. Second, the negatively polar N2 component and the subsequent positively polar P3 component occur approximately 200–500 ms post-stimulus onset (Liddell et al., 2004). The N2 component has been directly linked to the amygdala and the anterior cingulate cortex (Gläscher and Adolphs, 2003), and the P3 component has a posterior topography. Future research should use neuroimaging to explore the difference of acquired disgust or acquired fear signals in the extinction paradigm. And in order to improve ecological validity, it may be better to use more naturalistic stimuli such as fearful or disgust movies, music, and stories as acquired fear or acquired disgust to confirm the findings. Third, the subjects of this study are healthy college students. In order to provide theoretical support for clinical treatment, future research can take patients with different subtypes of OCD as subjects to study their differences of acquired disgust or acquired fear signals in the extinction paradigm.

## Data Availability Statement

The raw data supporting the conclusions of this article will be made available by the authors, without undue reservation.

## Ethics Statement

The studies involving human participants were reviewed and approved by Ethics Committee of School of Psychology, South China Normal University. The patients/participants provided their written informed consent to participate in this study.

## Author Contributions

QZ organized the data collection, performed the statistical analysis, wrote the manuscript, and contributed substantially to writing of the method and results section. XZ conceptualized the study and studied setup. LL was responsible for the language revision and arrangement of the article. All the authors contributed to the article and approved the submitted version.

## Conflict of Interest

The authors declare that the research was conducted in the absence of any commercial or financial relationships that could be construed as a potential conflict of interest.

## Publisher’s Note

All claims expressed in this article are solely those of the authors and do not necessarily represent those of their affiliated organizations, or those of the publisher, the editors and the reviewers. Any product that may be evaluated in this article, or claim that may be made by its manufacturer, is not guaranteed or endorsed by the publisher.

## Footnotes

1 IAPS identify numbers for disgust pictures:1270, 1271, 1274, 1275, 1280, 2710, 2730, 2750, 3019, 3140, 3160, 3250, 4635, 7359,7360, 7361, 7380, 9008, 9031, 9043, 9140, 9180, 9181, 9185, 9290, 9291, 9295, 9300, 9301, 9302, 9320, 9322, 9325, 9326, 9330, 9340, 9341, 9342, 9373, 9433, 9520, 9561,9570, 9571, 9830 and for fear pictures:1019, 1050, 1052, 1113, 1120, 1302, 1321, 1525, 1726, 1930, 1932, 2811, 3500, 5970, 5971, 6230, 6231, 6250, 6260, 6263, 6300, 6312, 6313, 6350, 6370, 6510, 6550, 6555, 6560, 6563, 6571, 6821, 7640, 8480, 9403, 9413, 9424, 9524, 9600, 9622, 9623, 9908,9920, 9940 and for neutral pictures:1450, 2036, 2102, 2377, 2383, 2393, 2396, 2411, 2560, 2850, 5471, 7000, 7001, 7002, 7003, 7004, 7006, 7009, 7010, 7021, 7035, 7041, 7045, 7050, 7053, 7056, 7057, 7059, 7061, 7080, 7081, 7090, 7100, 7150, 7175, 7185,7190, 7205, 7211, 7217, 7224, 7233, 7513, 7547, and 7705.
